# Bioactivity of arid region honey: an in vitro study

**DOI:** 10.1186/s12906-017-1664-9

**Published:** 2017-03-29

**Authors:** Serene Hilary, Hosam Habib, Usama Souka, Wissam Ibrahim, Carine Platat

**Affiliations:** 1 Nutrition and Health Department, College of Food and Agriculture, United Arab Emirates University, P. O. Box 15551, Al Ain, United Arab Emirates; 2 Alexandria University, 22 El-Guish Road, El-Shatby, Alexandria, 21526 Egypt

**Keywords:** Honey, Arid region, Antioxidant, Anti-inflammation, Anti-tumor, MDA, Hemolysis

## Abstract

**Background:**

Antioxidant and anti-inflammatory properties of honey have been largely recognized by various studies. Almost all of the potential benefits are associated with polyphenol content. Honey varieties from the arid region are reported to be rich in polyphenols, but data related to its bioactivity in vitro is greatly lacking. This study aimed at establishing the antioxidant and anti-inflammatory properties of arid region honey. Four honey varieties from arid region (H1, H2, H3, and H4) and two popular non-arid region honey (H5 and H6) were tested in vitro in this study.

**Methods:**

The erythrocyte membrane protection effect of honey varieties were measured by hemolysis assay after exposing erythrocytes to a peroxide generator. The subsequent production of MDA (malondialdehyde) content in erythrocytes was measured. Immunomodulatory effect of the honey varieties was tested in prostate cancer cells PC-3 and PBMC (peripheral blood mononuclear cells) by measuring the IL-6 (interleukin 6) and NO (nitric oxide) levels in cell culture supernatant after incubation with the honey varieties. PC-3 cell viability was assessed after incubation with honey varieties for 24 h.

**Results:**

Arid region honey exhibited superior erythrocyte membrane protection effect with H4 measuring 1.3 ± 0.042mMTE/g and H2 measuring 1.122 ± 0.018mMTE/g. MDA levels were significantly reduced by honey samples, especially H4 (20.819 ± 0.63 nmol/mg protein). We observed a significant decrease in cell population in PC-3 after 24 h in culture on treatment with honey. A moderate increase in NO levels was observed in both cultures after 24 h at the same time levels of IL-6 were remarkably reduced by honey varieties.

**Conclusion:**

The results demonstrate the antioxidant effect of arid region honey due to its erythrocyte membrane protection effect and subsequent lowering of oxidative damage as evident from lower levels of lipid peroxidation byproduct MDA. Arid region honey varieties were as good as non-arid region types at decreasing cell viability of prostate cancer cells. The moderate increase in NO levels in PC-3 and PBMCs were not significant enough to elicit any pro-inflammatory response. However, IL-6 secretion was remarkably reduced by all honey varieties in a comparable level indicating the potential anti-inflammatory property of arid region honey.

## Background

Honey is a viscous liquid produced by bees from the nectar of flowers. It is considered as a wholesome natural food and is also put to medicinal use. Honey has been shown to have broad spectrum anti-microbial activity against gram positive and gram negative bacteria [1] and it is also reported to have wound healing capabilities [2]. Many aspects of honey’s healing properties have been identified, including antioxidant, anti-inflammatory and anti-tumor properties [2, 3]. These health properties are of great interest since oxidative stress and inflammation are mechanisms involved in the development of major chronic diseases like cancer [4]. A strong relationship between oxidative stress and inflammation is highly suspected, where the inflammatory process could be activated by a pro-oxidant condition which yet again triggers the release of additional ROS (reactive oxygen species) [4, 5]. Chronic diseases are increasing throughout the world and a marked increase in chronic disease has been identified in the Arab Gulf Region [6].

Oxidative stress is defined as an imbalance between the production of free radicals and reactive metabolites, so-called oxidants or ROS. Protective mechanisms during metabolic reactions involve antioxidants which act mainly as ROS scavengers. The imbalance between oxidative stress and antioxidant scavengers leads to damage of important biomolecules in the cells. Lipids are one biomolecule that represent a significant target for ROS attack. The lipid peroxidation generates other reactive species such as MDA and can increase the risk of mutagenesis. Cancer initiation and progression have been linked to oxidative stress by increasing DNA mutations or inducing DNA damage, genome instability, and cell proliferation [7]. ROS scavenger activity and improvement of the antioxidant status are some of the mechanisms by which honey could exert its antioxidant action [8].

Inflammation is another condition which has been related to the etiology and the development of cancer. Prostate cancer is expected to be one of the major disease in males in the coming decades [6]. There is growing evidence for an implication of chronic inflammation in the origin of many cancers including prostate cancer. Inflammation leads to the release of pro-inflammatory cytokines, particularly IL-6 and NO. They are shown to contribute to the damage of macromolecules and cells, disruption of the cell division process and the transformation of a normal cell to a tumor cell. IL-6 was clearly identified as a key cytokine implicated in cancers of prostatic tissue, breast tissue, and colorectal tissues [9, 10]. Recently, PBMCs, the active lymphocyte and monocyte fraction of the blood, were shown to be associated with prostate cancer development, through the secretion of pro-inflammatory cytokines most notably IL-6 [11].

A more complex and multifaceted role of IL-6 and NO related to inflammation has been identified. NO was shown to act as a pro-inflammatory or anti-inflammatory agent, depending upon the site of release and its concentrations [12]. There is growing evidence of an immunomodulatory effect of honey [13–15]. A modulation of IL-6 and NO secretion with honey treatment was observed through in vitro and in vivo studies [8]. Due to its action on the oxidative balance and inflammatory process, honey was shown to modulate the initiation and the progression of many types of cancer among which breast, liver, and colon cancer have been extensively studied [8, 16, 17]. In spite of very promising data, honey’s effect on prostate cancer remains to be better explored.

It is now recognized that almost all of the biological activity of honey is credited to its composition [18]. This natural product has a complex chemical composition. All honey types are a mixture of sugars, bee proteins, vitamins, minerals, and polyphenols. Most of the antioxidant benefits of honey are associated with the presence of polyphenols [19]. There is a high correlation between honey’s biological activity and the polyphenolic content. Polyphenols are secondary metabolites of plants that act as dietary antioxidants in the body. More than 8000 compounds have been identified hitherto, and some of them like curcumin and catechins are extensively researched for potential therapeutic applications. The common polyphenols reported in honey include gallic acid, catechins, epicatechins, chlorogenic acids, caffeic acids, coumaric acids and quercetin [20]. The content of these polyphenols is different depending on the source and floral origin of honey. The climate where honey is made also significantly influences the polyphenol content and profile. Notably, it was demonstrated that some arid region honey possess a greater amount of polyphenols compared to famous non-arid region honey [21, 22]. Knowing this, it is reasonable to expect from arid region honey greater health benefits. Habib et al. [21] reported that one or more types of honey from arid regions presented a higher antioxidant potential compared to non-arid region honey varieties. Habib et al. [21] compared non arid region honey varieties such as Black Forest and Manuka to arid region honey by using biochemical assays. Non-arid region varieties of honey such as Manuka [23], Gelam [16] and Tualang [24] have been extensively studied in regards to their antioxidant, anti-inflammatory and anti-tumor properties. Results from these studies were promising. However, arid region honey in spite of their high polyphenolic content and greater antioxidant effect, which are both in favor of an anti-tumor effect, remain uninvestigated for their biological activity. Thus further exploration of the health properties of arid region honey is warranted. The aim of this research is to investigate the potential antioxidant, anti-inflammatory, and anti-tumor properties of some of the most popular varieties of arid region honey, and to compare arid region honey to popular non-arid region honey varieties.

## Methods

### Materials

Histopaque-1077, PBS (phosphate buffered saline), AAPH (2, 2’-azo-bis (2-amidinopropane) dihydrochloride), Trolox, and Trypan blue solution from Sigma-Aldrich; RPMI 1640, FBS (fetal bovine serum), and Penicillin-Streptomycin cocktail from Gibco; Bovine Hypothalamus Extract prepared in-house as per the method described by Maciag et al. [25]; Griess Reagent Kit for Nitrite Determination from Molecular Probes; IL-6 ELISA Kit from Novex, Life Technologies.

### Honey varieties

All honey varieties used for the study were purchased from local supermarkets. Honey varieties were selected for in vitro analysis based on the results reported by Habib et al. [21]. Four honey varieties were selected from the arid region, two monofloral (H1 and H3) and two heterofloral (H2 and H4), to compare with two non-arid region varieties H5 (monofloral) and H6 (heterofloral). All details about each of these honey types are described in Table 1. For hemolysis and MDA assay, honey varieties were dissolved in PBS to make 10% solution. For anti-inflammatory studies and cell viability test, 10% honey solution was prepared by dissolving in serum free media. The honey solutions were centrifuged at 3000 × g for 10 min to remove any undissolved particles and filtered using 0.45 μm membrane filter.

**Table 1:** Description of honey varieties by region

Sample	Type	Family	Botanical name	Common name	Country
Arid region					
H1	Monofloral	Rhamnaccae	*Ziziphus spina-csisti*	Wild jujube	United Arab Emirates
H2	Heterofloral	Fabaceae	*Acacia tortilis*	Wild mountain	Yemen
H3	Monofloral	Fabaceae	*Acacia tortilis*	Wild mountain	United Arab Emirates
H4	Heterofloral	Fabaceae	*Acacia tortilis*	Mountain herbal	Yemen
Non arid region					
H5	Monofloral	Myrtaceae	*Leptospermum scorparium*	Manuka	New Zealand
H6	Heterofloral	Myrtaceae	*Leptospermum scoparium*	Black forest	Germany

### Hemolysis assay

Erythrocyte membrane protection effect of the honey varieties was measured using the method described by Banerjee et al. [26] with some modifications. Briefly, whole blood was collected from a healthy donor in EDTA tubes and was centrifuged at 750 × g for 5 min at room temperature to separate the platelet rich plasma. The erythrocytes were washed twice with PBS, and a 5% suspension of the erythrocytes was prepared in PBS for the assay. 500 μl of the 5% erythrocyte suspension was exposed to 200 μl of 10% honey samples (v/v in PBS) or 200 μl of Trolox standards for 30 min at 37 °C. AAPH was added to the samples to attain a concentration of 50 mM in solution, and the tubes were further incubated for further 2 h at 37 °C. The samples were centrifuged at 3000 × g for 10 min and supernatant was collected. The absorbance of the supernatant was measured using a UV-Visible spectrophotometer at wavelength 540 nm. The experiment was carried out in triplicates and data were expressed as mM Trolox Equivalent (TE)/g.

### MDA assay

The decrease of MDA content by honey in erythrocytes after incubation with peroxide generator AAPH was measured by TBARS (Thiobarbituric acid reactive substrates) method described earlier by Seljeskog et al. [27] with some modifications. 500 μl of 5% erythrocyte suspension prepared in PBS was incubated with 200 μl of honey varieties H1, H2, H3, H4, H5, H6 (10% v/v in PBS) or 40 mM Trolox (positive control) or PBS. After 30 min incubation AAPH was added to the samples to attain a concentration of 50 mM in solution and the mixture was incubated for 2 h at 37 °C. The control used for the experiment was erythrocyte suspension incubated with PBS and treated with AAPH. Following incubation, an equal volume of deionized water was used to lyse the cells. Erythrocyte suspension was directly lysed with deionized water to measure the native MDA content in the cells. The samples were then subjected to protein precipitation using 20% TCA and centrifuged at high speed to separate the protein. The supernatant was aliquoted to new tubes and were incubated with 0.4% TBA (Thiobarbituric acid, w/v in 0.2 N HCl) at 60 °C for one hour for the formation of TBA-MDA adducts. The TBA-MDA adduct formed in the sample was measured by Breeze HPLC System (Waters, USA). The mobile phase was methanol:0.05 M KH_2_PO_4_ buffer pH 6.8 (40:60, v/v) containing 0.2% (v/v) triethanolamine. Xterra MS C18 reverse phase column of 5 μm pore size was used for the analysis. The column temperature was 35 °C, and the flow rate was 1 ml/min with an injection volume of 20 μl. The fluorescence detection wavelength was set at 532 nm (excitation) and 553 nm (emission). All samples were analysed in triplicates.

### PBMC isolation and culture

PBMCs were isolated from whole blood using density gradient centrifugation in Histopaque-1077 as per manufacturer’s protocol. Briefly, blood samples were obtained from healthy donors in EDTA tubes and diluted with equal volume of PBS and layered over Histopaque and centrifuged at 400 × g for 30 min at room temperature in a bench top centrifuge. The layer containing PBMCs were carefully separated from the Histopaque interface and washed twice with serum-free RPMI 1640 medium by centrifuging at 250 × g for 10 min. The washed PBMCs were resuspended in RPMI 1640 substituted with 10% FBS, 100u/ml penicillin, 100 μg/ml streptomycin and 25 μg/ml bovine hypothalamus extract and maintained at 37 °C in 5% CO_2_ incubator.

### PC-3 culture

Prostate cancer cell line, PC-3 was purchased from European Collection of Authenticated Cell Cultures (ECACC), UK and was cultured in RPMI 1640 medium substituted with 10% FBS, 100U/ml Penicillin and 100 μg/ml Streptomycin. Upon reaching 80% confluence, the cells were passaged using 0.25% Trypsin.

### Anti-inflammatory properties

PC-3 cells and PBMCs were seeded at a density of 4 × 10^5^ cell/ml in six-well plates, and grown to 80% confluency in complete growth medium (RPMI 1640 substituted with 10% FBS, 100u/ml penicillin, 100 μg/ml streptomycin and 25 μg/ml bovine hypothalamus extract for PBMCs and RPMI 1640 medium substituted with 10% FBS, 100U/ml Penicillin and 100 μg/ml Streptomycin for PC-3). Upon reaching 80% confluency, the cells were starved overnight with serum-free media and subsequently medium substituted with 10% honey solution was introduced to cells. The culture was incubated for 24 h at 37 °C in 5% CO_2_ incubator, following which the culture supernatant was retrieved for the experiments. The IL-6 and NO levels in PC-3 and PBMC culture supernatants were measured by commercial ELISA kit and Griess reaction method respectively. Untreated cells grown in respective complete growth medium served as control for the experiments. All experiments were carried out in triplicates.

### Viability test

Ten percent honey samples were prepared in serum-free media and filter sterilized. PC-3 cells were seeded at a density of 4 × 10^5^ cell/ml in six-well plates, upon reaching 80% confluency, the cells were starved overnight with serum-free media following which medium containing 10% honey was introduced to cells. The culture was incubated for 24 h at 37 °C in 5% CO_2_ incubator. Post incubation the viability was assessed by trypan blue exclusion assay, and percentage viability was calculated. Experiments were carried out in triplicates, and untreated cells grown in complete growth medium was used as control.

### Statistical analysis

All data are presented as mean ± SD. Statistical analysis was done by Post Hoc Tukey Test using SPSS statistical software version 20 for all quantitative parameters to identify the significance of the difference between control and test groups. *P*-value of <0.05 was considered as statistically significant.

## Results

### Hemolysis assay

Membrane protection effect was used to demonstrate antioxidant activity of the honey varieties. Table 2 shows the results from the hemolysis assay. In this assay, peroxide generator AAPH was added to cells to induce lysis and decrease in hemolysis by honey treatment was measured in terms of trolox equivalents. All honey varieties were found to be effective in protecting the erythrocyte membrane from oxidative damage. In this study, H4 showed the highest antioxidant activity at 1.3 ± 0.042mMTE/g. This is significantly higher than all other samples except H2. Sample H2 was second in effectively preventing hemolysis indicating a higher antioxidant effect at 1.122 ± 0.018mMTE/g. Interestingly, the antioxidant potential of both H2 and H4 was significantly higher than both non-arid region types H5 and H6, measuring 0.907 ± 0.053mMTE/g and 0.668 ± 0.040mMTE/g respectively. The lowest measured antioxidant effect which stood at 0.668 ± 0.040mMTE/g was observed in H6. In comparison H1 and H3 showed significantly higher erythrocyte membrane protection effect than H6, with 1.046 ± 0.089mMTE/g and 1.014 ± 0.044mMTE/g, respectively.

**Table 2:** Anti-hemolytic effect of honey varieties (mM Trolox Equivalent/g)

Samlple	mM Trolox Equivalent/g
H1	1.046 ± 0.089 ^a^
H2	1.122 ± 0.018 ^b^
H3	1.014 ± 0.044 ^c^
H4	1.30 ± 0.042 ^d^
H5	0.907 ± 0.053
H6	0.668 ± 0.040 ^e^

Means ± s.e. are presented

^a^Significant difference between H1 and H4

^b^ Significant difference between H2 and H5

^c^Significant difference between H3 and H4

^d^Significant difference between H4 and H5

^e^Significantly lower than all honey varieties

### MDA assay

**Fig. 1:**
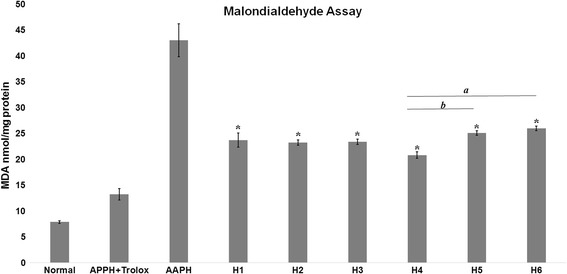
Malondialdehyde content measured by TBARS assay in erythrocytes after treatment. Data expressed as nmol MDA/mg protein. Normal represented the amount of MDA in erythrocytes without treatment. Normal and AAPH + Trolox were used as positive controls for the experiment. Comparisons using Post Hoc Tukey test was carried out for AAPH, H1, H2, H3, H4, H5, and H6. *Significant decrease in MDA levels compared to AAPH treated group. *a* - Significant difference in MDA levels between H4 and H6. *b* - Significant difference between H5 and H6

All honey varieties significantly reduced the amount of MDA content formed as a result of peroxyl radicals generated by AAPH (Fig. 1) when compared to the control group (AAPH in Fig. 1). The positive control of the experiment, Trolox, in combination with AAPH, showed a significant lowering of MDA levels with 13.23 ± 1.12 nmol/mg protein. This was significantly lower than the control and all honey varieties. Arid region honey H4 demonstrated the overall best antioxidant effect with a measured MDA level of 20.819 ± 0.63 nmol/mg protein which indicates its ability to lower oxidative damages than both non-arid region varieties H5 (25.11 ± 0.42 nmol/mg protein) and H6 (25.98 ± 0.44 nmol/mg protein). All other honey varieties presented a comparable effect in reducing oxidative damage to erythrocytes.

### Nitric oxide levels

**Fig. 2:**
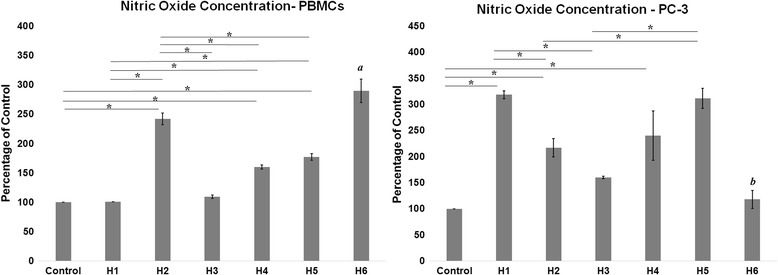
Nitric oxide concentration after 24-h incubation with honey varieties. Data expressed as percentage of control. *Significant difference in NO levels observed between groups. *a* - Significant increase of NO levels in H6 treated groups in comparison to all honey varieties and control in PBMCs. *b* - Significant lowering of NO level in H6 treated groups in comparison to H1, H2, H4, and H5 in PC-3

Nitric oxide level in both PBMC and PC-3 cultures are presented in Fig. 2. The levels detected in PBMCs were significantly higher in all honey varieties except H1 and H3 in which percentage detected was similar to control group. Among the arid region honey, the percentage of NO in H2 was the highest at 242.03% ± 9.97%. The level of NO in H4 treated group in PBMCs was also increased at 160.09% ± 3.58%. Interestingly, both H2 and H4 are heterofloral varieties. PBMCs elicited the highest response in H6 group, which was significantly higher than all other groups, in which the measured percentage of NO level was 289.70% ± 19.99%. Only H2 in the arid region had a comparable effect in PBMCs as seen in H6. The effect of H5 on PBMCs measuring 177.17% ± 5.64% was only slightly higher than H4. However, NO levels in H1 and H3 was much lower than H5. PC-3 culture showed the remarkable result in NO production in treatment with the honey samples. The effect of each honey type in the PC-3 cell was different from that of PBMCs. Here, all groups except H3 and H6 gave a significantly higher increase in NO production when compared to control. Unlike the response in PBMCs, H1 recorded the highest percentage increase in NO at 318.16% ± 7.38% and H6 recorded the least increase at 117.94% ± 17.32%. The level of NO increase seen in H1 groups was similar to H5 which stood at 311.47% ± 19.38%. This is clear indication that arid region honey H1 has a comparable effect to that of non-arid region honey H5. The effect of H2 (216.39% ± 17.71%) and H4 239.89% ± 47.14%) was however significantly better than that of H6 non-arid honey. The response from H3 (159.55% ±2.3%) measured was higher than control and H6 although it was not significant.

### IL-6 ELISA

**Fig. 3:**
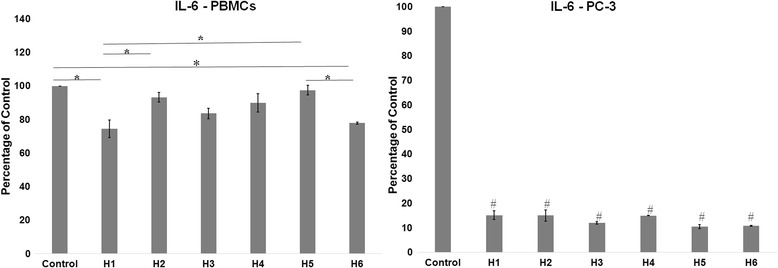
IL-6 concentration after 24-h incubation with honey varieties. Data expressed as percentage of control. *Significant difference in IL-6 concentration observed between treatment groups in PBMCs. # Significant lowering of IL-6 concentration in honey treated groups compared to control in PC-3 cells

IL-6 secretion by PBMCs and PC-3 cells were lowered when compared to control on treatment with the honey (Fig. 3). The percentage of IL-6 secretion in PBMCs, however, was significantly lowered only in H1 (74.56% ± 5.25%), H3 (83.57% ± 4%) and H6 (77.87% ± 0.66%). Arid region honey H1 elicited the most effective reduction in PBMCs, and the measured percentage was comparable with non-arid region variety. Meanwhile, H5 (97.49% ± 2.93%) gave the least effect in the study. The response induced by H2 (93.23% ± 2.8%) and H4 (89.92% ± 5.4%) from the arid region markedly better than H5, however, were not significant. From arid region honey, the monofloral varieties (H1 and H3) elicited great reduction when compared to a similar variety from the non-arid region (H5). The percentage secretion of IL-6 by PC-3 cells was considerably lowered in comparison to control. In all groups, the data was significantly lower than the control. All honey varieties showed a similar effect in lowering the IL-6 secretion from PC-3, there were not significantly different from each other.

### Cell viability

**Fig. 4:**
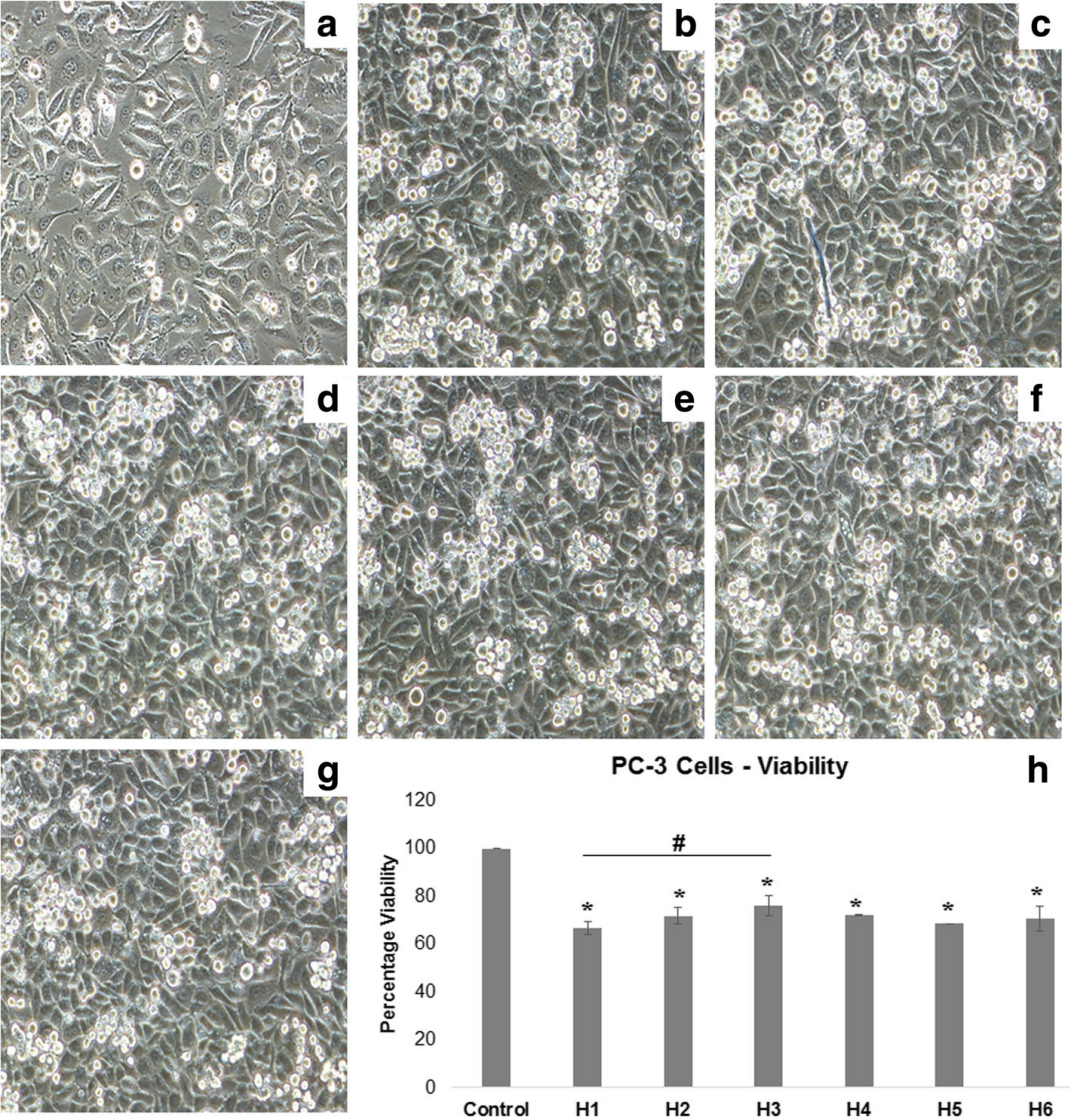
Viability of PC-3 cells after 24-h incubation with honey. **a** Untreated Control, **b** H1, **c** H2, **d** H3, **e** H4, **f** H5, **g** H6. Cell under 20X magnification. **h** Percentage decrease in viability of cells after 24-h incubation with honey varieties. *Treatment groups shows significant lowering of viability compared to control. # - Significant difference between H1 and H3

After 24-h incubation with honey varieties, there was a marked difference in the morphology of the PC-3 cells treated with honey compared to that of the control (Fig. 4a–g). The honey-treated group appeared to be more rounded and shrunk in size compared to the untreated group which appeared elongated and stretched which is characteristic to PC-3 cells. The viability of the cells in the honey treated group was found to be significantly lower compared to the control (Fig. 4h). The viability of PC-3 cells was effectively reduced by all arid region varieties. H1 was the most effective honey variety in lowering viability to 66.89% ± 2.76% which was significantly better than H3 which has the least capacity in lowering viability measuring 76.08% ± 4.4%.

## Discussion

This in vitro study investigated the antioxidant, anti-inflammatory and anti-tumor properties of the popular honey varieties from the arid region. For the study, two monofloral (H1 and H3) and two heterofloral (H2 and H4) varieties of honey were selected from arid region to compare with popular monofloral and heterofloral varieties from the non-arid region, namely Manuka and Black Forest (H5 and H6, respectively). Globally, antioxidant, anti-inflammatory and anti-tumor effects were demonstrated for arid region honey. Arid region honey showed a greater antioxidant effect than non-arid honey as shown by the prevention of oxidative damages to erythrocytes. The variety H4, a heterofloral mountain herbal variety from Yemen, was the most effective in lowering erythrocyte lysis by peroxide radicals whereas all other arid region varieties showed comparable effect to non-arid region varieties. Reduction of lipid peroxidation as seen by lowering of MDA levels by H4 also indicates its superior ability is lowering oxidative damage. Decrease in IL-6 level was observed, in a similar way, in arid and non-arid region honey indicating comparable anti-inflammatory effect of the varieties. One of the most significant results noted was the reduced viability of PC-3 cells on treatment with the honey varieties, indicating its potential anti-tumor effect.

The free radical-induced erythrocytes hemolysis is recognized as a particularly efficient method for evaluation of antioxidant capacity, with some advantages compared to common biochemical assays like TAC or DPPH. It uses free radicals as pro-oxidants and erythrocytes as oxidizable targets so that the results obtained reflect biologically relevant radical-scavenging activity. Lysis of erythrocytes, which transport oxygen and are one of the most abundant cells in the human body, is related to oxidative stress. Free Radicals, generated by this oxidative stress, are known to attack highly unsaturated fatty acids to induce lipid peroxidation, leading to changes in the fluidity of the cell membrane and ultimately to cell injury and lysis. Erythrocytes are more likely to be exposed to this type of oxidative damage due to high lipid content in their membranes and presence of transition metals like iron and copper. Arid region honey demonstrated a protection effect to erythrocytes against lysis by free radicals generated by AAPH. Similar results were observed in an in vivo study where enhanced cytoprotective function was demonstrated against doxorubicin toxicity in mice supplemented with Seder mountain honey [28].

Also, arid region honey was able to protect against lipid peroxidation in erythrocytes. Erythrocytes hemolysis is related to oxidative damages on lipid bilayer. One of the principle markers of oxidative damage in erythrocyte is the lipid peroxidation product MDA. An increase in MDA level will be observed in cells that are exposed to free radicals. Therefore, an effective lowering of MDA levels by honey varieties supports its antioxidant property. While such an antioxidant effect had already been shown by Habib et al. [21], in arid region honey, it was done by using common biochemical assays (DPPH, FRAP, TAC) and not cell-based tests.

The anti-inflammatory effect of honey was long established through various studies [14, 16, 23, 29] on non-arid region honey, yet nothing was available on arid region varieties. The research results demonstrated here indicate that arid region honey also possesses anti-inflammatory effects similar to previously studied non-arid honey types. This is demostrated by its capacity to lower IL-6 in prostate cancer cell line and PBMCs. IL-6, as an early signal, is a key component in the inflammatory process. It is secreted by T cells and macrophages to stimulate inflammation at the time of infection or tissue damage. It is noteworthy that all honey types were equal in their ability to lower the cytokine level in both cell types. In PBMCs, the decrease in IL-6 levels, however, was not as remarkable as PC-3. This can be related to the fact that prostatic cancer tissue secretes cytokine IL-6 in great quantities and that PBMCs were under normal conditions. As reported earlier expression of IL-6 is associated with the development of prostate cancer through an immunomodulatory dialog with cancer cells [11]. The result observed in this study takes precedence as it clearly demonstrates an immunomodulatory effect in prostate cancer cell line. Considering that health properties of honey were related to their polyphenol content, the result of this study are in accordance with the recent study in which treatment with polyphenols were able to lower the expression of pro-inflmmatory cytokines in PBMCs [30].

Surprisingly, where an anti-inflammatory effect of honey could be suggested based on the data of IL-6, the effect of honey on NO production was apparently in favor of a pro-inflammatory action. Indeed, it was found that all honey varieties were capable of increasing the NO levels in varying degrees in PC-3 cells and PBMC, which accounts for initiation of systemic pro-inflammatory response. NO, which is an important mediator of inflammation, is a double-edged compound, playing a paradoxical role depending upon the concentration at which it is secreted by cells. Typically, under low physiological concentration, NO acts as an anti-inflammatory molecule, but at higher concentration it acts as a mediator of inflammation [12]. Here, the increase of NO production was least in both cultures on treatment with arid region variety H3. On the contrary, the non-arid region types gave varying data in both cultures, where H6 gave a significant increase of NO in PBMCs while the increase was minimal in PC-3 cells. However in both cell types studied, an increase of NO by a 0.6–2 μM from the control concentration was observed, this cannot be significant enough to be considered pro-inflammatory as it is reported that a 100 fold increase is at least observed at the time of pro-inflammatory signaling by NO [31, 32]. Therefore, it seems that the increase of NO levels which was detected in our study was not strong enough to elicit any pro-inflammatory response.

Finally, since oxidative stress and inflammation are two major mechanisms involved in the etiology and development of cancer [4], the research results identified here indicate that arid region honey could effectively regulate growth and metastasis of prostate cancer cells. This is supported by our result which have shown that IL-6 was reduced with supplementation of honey in PC-3 cells. IL-6 is associated with aggressive prostate cancer phenotype and may be involved in the metastatic process through regulation of epithelial-mesenchymal transition and homing of cancer cells to the bone [9]. It is also interesting to note that in vivo studies in animals and human clinical trials have been conducted with anti-IL-6 monoclonal antibodies to target prostate tumor [33]. This would mean that any factor able to reduce the secretion of IL-6 could exert an anti-tumor action. In addition, we found the viability of PC-3 cells was reduced on treatment with honey for 24 h. The capacity of honey observed in this study, (both arid and non-arid region varieties) to significantly reduce the population of PC-3 cells and reduce its capacity to secrete IL-6, enables us to suggest a possible anti-tumor effect for honey in prostate cancer.

A tight inter-relationship between oxidative stress and inflammation is now well acknowledged [34]. However, the sequence of these events remains unclear and impedes the identification of therapeutic targets. Some studies clearly showed that one of the important aspects of inflammation is the sudden increase in the superoxides, also called oxidative burst, as a result of phagocyte activation. Also, it was observed that heme released by erythrocytes as a result of lysis lead to neutrophil migration and triggered oxidative burst by these cells [35]. The capacity of antioxidants to protect the integrity of the cell membrane was found to be a very critical event in preventing inflammation, indicating that oxidative damages would be the one to induce the inflammatory process. The present study suggest that honey increased protection of erythrocytes against lysis through the reduction of lipid peroxidation. This supports the theory of the antioxidant effect in honey.

Globally, our results are significant in the context that accumulation of oxidative damages in the body can later result in the development of most chronic diseases. The erythrocyte membrane protective function of honey can effectively reduce the risk of inflammation from hemolysis which has been implicated in several diseases leading to organ damage. For example, the cytoprotective effect of Seder honey variety, from the arid region, was assessed in doxorubicin-treated mice [28]. In this study, enhanced liver function and improvement in symptoms such as bleeding were demonstrated in the treated groups.

In view of the fact that polyphenols in honey are reported to have antioxidant and anti-inflammatory effects [36–38], it can be reasonably assumed that similar results would occur using the honey varieties from arid region which have high polyphenol content. A more comprehensive study on the polyphenol content of these varieties would demonstrate which molecules are directly involved in bringing about this effect. An earlier study by Habib et al. [21] had probed more than ten arid region varieties against non-arid region types showing the polyphenol content in the honey were comparable and in some varieties even better than non-arid region varieties. Greater health effects of arid region honey would be expected if a higher polyphenol content is present. Here, under in vitro conditions, the antioxidant potential of arid region honey was either comparable or stronger, in case of H4, than non-arid region varieties. The anti-inflammatory and anti-tumor properties of the arid region were found to be comparable to non-arid region types.

This work is presenting some limitations. One limitation was related to the concentration of honey and the time of cell exposure to honey. This could be addressed by probing the different concentrations of honey varieties used in this study (concentration of honey used in our study was 10% which was calculated based on results reported from the study by Habib et al.) and by assessing the long-term effect of honey supplementation in cell culture. Further experiments should be performed to clarify the anti-inflammatory role of arid region honey, including the study of additional cytokines like TNF-alpha, in PBMCs after inducing inflammation.

## Conclusions

Results obtained from our study has demonstrated that arid region honey is a potential antioxidant, anti-inflammatory and anti-tumor agent, as shown by the study of erythrocyte lysis, erythrocyte membrane protection against oxidative damages, cancer cell growth inhibition, and reduction of pro-inflammatory marker secretion in PC-3 and PBMC culture. Compared to non-arid region varieties, arid region honey showed a greater antioxidant potential and comparable anti-inflammatory and anti-tumor effect. An immunomodulatory effect of arid region and non-arid region honey was further suggested. All of this is in favor of a promising use of arid region honey as a therapeutic product for major chronic diseases, especially cancer.

## Abbreviations

AAPH: 2, 2’-azo-bis (2-amidinopropane) dihydrochloride
FBS: Fetal bovine serum
IL-6: Interleukin-6
MDA: Malondialdehyde
NO: Nitric oxide
PBMC: Peripheral blood mononuclear cells
PBS: Phosphate buffered saline
ROS: Reactive oxygen species
TBA: Thiobarbituric acid
TBARS: Thiobarbituric acid reactive substance
